# Conscious expectancy rather than associative strength elicits brain activity during single-cue fear conditioning

**DOI:** 10.1093/scan/nsad054

**Published:** 2023-09-26

**Authors:** Laurent Grégoire, Tyler D Robinson, Jong Moon Choi, Steven G Greening

**Affiliations:** Department of Psychology, Louisiana State University, Baton Rouge, LA 70803, USA; Department of Psychology and Brain Sciences, Texas A&M, College Station, TX 77843-4235, USA; Department of Psychology, Louisiana State University, Baton Rouge, LA 70803, USA; Department of Psychology, Louisiana State University, Baton Rouge, LA 70803, USA; Statistical Methodology Division, Statistics Research Institute, Daejeon 35208, South Korea; Department of Psychology, Louisiana State University, Baton Rouge, LA 70803, USA; Department of Psychology, University of Manitoba, Winnipeg R3T 2N2, Canada

**Keywords:** fear conditioning, expectancy, associative strength, dlPFC, fMRI, single-cue conditioning

## Abstract

The neurocognitive processes underlying Pavlovian conditioning in humans are still largely debated. The conventional view is that conditioned responses (CRs) emerge automatically as a function of the contingencies between a conditioned stimulus (CS) and an unconditioned stimulus (US). As such, the associative strength model asserts that the frequency or amplitude of CRs reflects the strength of the CS–US associations. Alternatively, the expectation model asserts that the presentation of the CS triggers conscious expectancy of the US, which is responsible for the production of CRs. The present study tested the hypothesis that there are dissociable brain networks related to the expectancy and associative strength theories using a single-cue fear conditioning paradigm with a pseudo-random intermittent reinforcement schedule during functional magnetic resonance imaging. Participants’ (n = 21) trial-by-trial expectations of receiving shock displayed a significant linear effect consistent with the expectation model. We also found a positive linear relationship between the expectancy model and activity in frontoparietal brain areas including the dorsolateral prefrontal cortex (PFC) and dorsomedial PFC. While an exploratory analysis found a linear relationship consistent with the associated strength model in the insula and early visual cortex, our primary results are consistent with the view that conscious expectancy contributes to CRs.

## Introduction

Fear is associated both with the conscious experience of feeling afraid and autonomic reactions, such as the skin conductance response (SCR) or heart rate changes, related to defensive behaviour (Lang *et al.*, 2000; Beckers *et al.*, 2013). While some research indicates that these aspects of fear are related (Kron *et al.*, 2013; Jiang *et al.*, 2021), other research indicates that the conscious experience of fear is dissociable from the autonomic response to fearful stimuli (Lang *et al.*, 2000; LeDoux and Pine, 2016).

Pavlovian conditioning is a prevalent procedure used to study fear in humans. This paradigm consists of repeated pairing of an initially neutral conditioned stimulus (CS+) with an aversive unconditioned stimulus (US; e.g. a mild electric shock). As a result, the presentation of the CS+ alone elicits a conditioned response (CR; e.g. self-reported fear or elevated SCR). Despite its apparent simplicity and extensive study (Lovibond and Shanks, 2002; Sehlmeyer *et al.*, 2009; Mertens and Engelhard, 2020), the cognitive and neural processes underlying fear conditioning, and Pavlovian conditioning more generally, are still a topic of much debate (Mitchell *et al.*, 2009; Mertens and Engelhard, 2020).

One common interpretation is that CRs emerge as a function of the associative strength between a CS and an US (Rescorla and Solomon, 1967; Perruchet, 2015), encoded in the amygdala independent of declarative knowledge of the association (Bechara *et al.*, 1995; Clark and Squire, 1998). This associative strength model posits that the magnitude of the CR changes as a function of the CS–US association history. For example, as the number of sequential CS + US (i.e. trials in which the CS and US are both presented) trials increases, the strength of the association increases, and conversely, the strength of the association decreases as the number of sequential CS trials without the US (i.e. CS-alone) increases. Previous research in both humans and non-human primates indicates that amygdala activity reflects the associative strength between the CS and US (Belova *et al.*, 2008; Li *et al.*, 2011), as does activity in the sensory regions associated with the CS (Weinberger, 2004; Moratti and Keil, 2009; Miskovic and Keil, 2012; Greening *et al.*, 2016; Yuan *et al.*, 2018).

The expectancy model offers an alternative perspective predicting that human fear conditioning is the result of a conscious expectation of the US following the presentation of the CS. According to this model, propositional knowledge of the association is required for the generation of a CR (Mitchell *et al.*, 2009; Boddez *et al.*, 2020). One example is the so-called gambler’s fallacy, in which sequential unreinforced CS+ trials increase participants’ expectation of shock despite a fixed reinforcement rate of 50% (Burns and Corpus, 2004), which is associated with the lateral prefrontal cortex (Xue *et al.*, 2012). Another example is instructed fear conditioning in which being told the CS–US relationship is sufficient to generate greater activity in the dorsal medial prefrontal cortex (dmPFC) during the conscious appraisal of differential conditioning according to a meta-analysis of functional magnetic resonance imaging (fMRI) studies (Mechias *et al.*, 2010). Although this meta-analysis did not find that the amygdala was reliably active in studies of instructed fear conditioning after correcting for multiple comparisons, several individual studies have reported amygdala activity (Phelps *et al.*, 2001; Olsson and Phelps, 2007). Other research suggests that the dorsolateral prefrontal cortex (dlPFC) is involved in participants’ conscious knowledge of the relationship between the CS and US (Carter *et al.*, 2006). These findings potentially reflect the role of parts of the dorsal prefrontal cortex, including dmPFC and dlPFC, in the anticipation of threat and the conscious experience of fear and anxiety (Harrison *et al.*, 2015; Taschereau-Dumouchel *et al.*, 2019). Additionally, human brain lesion research suggests parts of the dmPFC and dlPFC are involved in the conscious appraisal, though not the acquisition, of CRs (Kroes *et al.*, 2019).

**Fig. 1.**
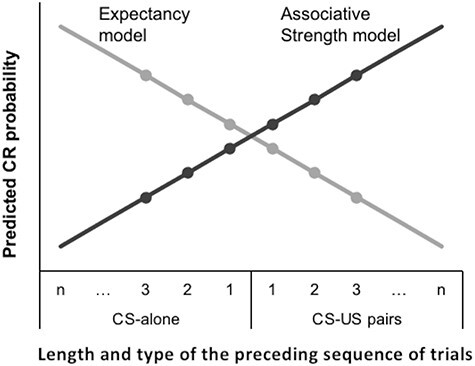
Visualization of the predicted hypothetical CR by the length of the preceding trial sequence for the Expectancy *vs* Associative Strength model.

In order to dissociate the predictions of the expectancy model from those of the associative strength model, the present study adopted a single-cue conditioning paradigm (Figure 1) combined with a pseudo-randomized trial sequence and a partial reinforcement schedule of 50% (Perruchet, 1985). The experiment comprised sequential CS + US or CS-alone trials of various lengths. This study is the first fMRI study to test the hypothesis that while activity supporting the associative strength model would manifest in the amygdala and sensory cortices, support for expectancy model would be observed in the dmPFC and dlPFC. The identification of dissociable networks contributing to each model would be consistent with contemporary two-system models of fear conditioning (LeDoux and Pine, 2016).

## Methods

### Participants

Twenty-three participants were recruited from the Louisiana State University community. All were English speakers and reported normal or corrected-to-normal visual acuity. No participant had neurological or psychiatric antecedents, and none were taking medication known to affect the central nervous system. One participant was excluded because of a technical issue. Data from one additional participant were discarded due to excessive head movements during the fMRI session. The final sample included 21 participants (11 females, mean age = 24.24 years, *SD* = 5.90). This study was approved by the Institutional Review Board of the Louisiana State University. All participants provided written informed consent prior to testing, being fully aware of the nature of the stimuli to be presented. Subjects received a financial compensation of $45 for their participation.

### Apparatus

The experimental paradigm was delivered using Matlab R2016b (Version 9.1; MathWorks, Natick, MA, USA) with the Psychtoolbox extension. Shock stimuli were administered using the STMISOC and STM100C modules of BIOPAC Systems and delivered by means of two MRI-safe electrodes placed on the distal phalanges of the fourth and fifth digits of the non-dominant hand. Participants reported shock expectancy by way of a two-response button box held in their dominant hand.

### Stimuli

The single face stimulus used in this study as the CS was a standardized 400 × 400 px greyscale face image from the Cohn-Kanade emotional faces database. The image depicts a white male with a neutral emotional affect. The image was cropped to show only the face from hairline to chin against a flat grey background. The US consisted of a mild 50 ms pulsed electric shock at 50 Hz (10 pulses with a duration of 5 ms each). Shock intensity was calibrated independently for each participant (see below for details). Face (15 male, 15 female) and place (15 house, 15 building) images used in the functional localizer task following the experimental sessions were drawn from the same standardized image database. All were emotionally neutral 400 × 400 images in greyscale against flat grey backgrounds. The CS used in the experimental sessions was not included in the localizer set.

### Design

**Fig. 2.**
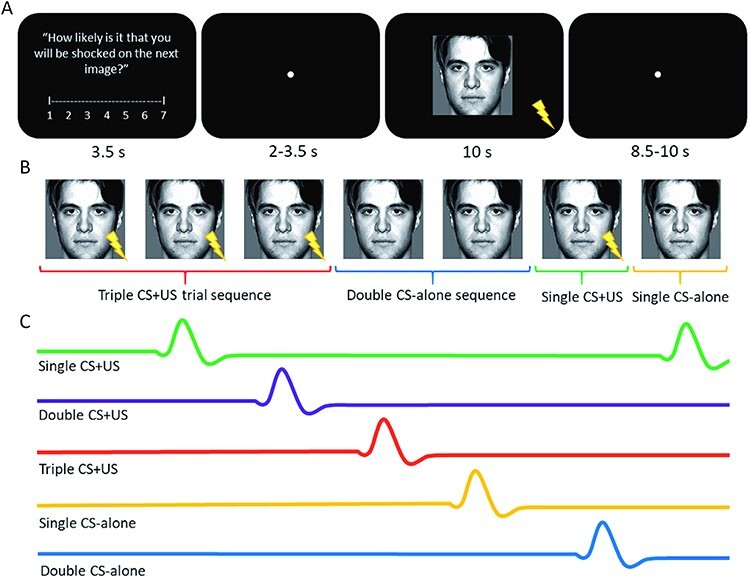
(A) Sample trial structure and (B) a visualization of hypothetical CS + US and CS-alone trial sequences. Note, in the trial sequences participants view the whole trial as exemplified above. Conditions are labelled and analysed relative to the preceding trial sequence, including sub-sequences within longer sets. The response functions in (C) depict hypothetical modelling of blood-oxygenation-level-dependent (BOLD) signal by trial in order to separate sequence types, i.e. the trial following two CS + US images is labelled ‘double CS+US.’.

The experimental design was based on the one used by Perruchet (1985) and Moratti and Keil (2009). The main task consisted of three runs (i.e. functional runs in the scanner) of 45 trials per run for a total of 135 trials across all three runs. Half the trials were CS-alone trials in which the US was omitted. In the other half of the trials (CS–US pairs), the CS co-terminated with the US. In each run, the 45 trials were presented as sequences of one-in-a-row (single), two-in-a-row (double) or three-in-a-row (triple) CS + US trials and sequences of one-in-a-row (single), two-in-a-row (double) or three-in-a-row (triple) CS-alone trials (Figure 2B). The sequences were designed to conform to a binomial distribution of two equally probable events (CS-alone trials and CS + US trials). In a given run, the various sequences were randomly shuffled, following a method adopted from Nicks (1959). Additionally, a CS-alone trial was added at the end of each run to measure expectancy ratings and brain activity related to the last sequence of the run.

### Procedure

A graphical representation of the experimental procedure can be found in Figure 2A. Each trial began by presenting the Likert response task for 3.5s, followed by a variable inter-stimulus interval (ISI) of 2s to 3.5s with a fixation dot before presenting the CS image for 10s. Each trial was followed by a variable inter-trial interval (ITI) of 8.5s to 10s with fixation.

Before starting the main task, participants performed eight practice trials. They were instructed to pay attention to the face presented in each trial and to rate their expectancy for shock occurrence during the inter-trial interval. An explanation of the use of the rating device was provided prior to the practice session. Participants used a two-response button box to report their expectancy ratings. When they pressed on the left button, a marker on the screen moved toward the left side of a horizontal scale, and an opposite effect was obtained by pressing on the right button. The rating scale on the screen was divided into seven levels, from ‘1 = not at all’ to ‘7 = very much so’. When the scale appeared on the screen, the cursor was always pointed at ‘4’, which meant that the subjective likelihood of receiving a shock on the next trial was 50%. The scale remained on the screen for 3.5 seconds (preliminary tests showed this time was sufficient to properly perform the expectancy rating task). Participants were informed that no shock was delivered in the practice, and the purpose was to become familiar with the task.

After the practice, electrodes for electrical stimulation were secured, and the shock level was adjusted individually to be ‘unpleasant but not painful’ (*M*_intensity_ = 5.91 mA, *SD* = 3.64, range: 1.60–20.00 mA; (Murty *et al.*, 2012; Grégoire and Greening, 2019, 2020). Instructions included the information that the presentation of the face was randomly followed by an electrical stimulation on 50% of the trials. Timing was consistent between the practice and experimental sessions. Experimental sessions consisted of 45 trials, the final trial in each session being a dummy presentation to allow time for Likert reporting of the previous presentation.

To independently localize face processing in the amygdala, a single functional localizer run was conducted. Standardized sets of 20 neutral face and 20 place images were drawn from the same Cohn-Kanade database as the experimental face stimulus. Images were divided into category-specific blocks made up of rapid event-related trial sequences. Each trial sequence involved the presentation of an image for 0.75s with a 0.25s ISI. Each block was presented five times in alternating order with a 15s ITI between face and place blocks.

### fMRI acquisition and analysis

#### MRI acquisition

BOLD data for the fMRI analysis were acquired using a 3 T GE Discovery MR750w research scanner with 36-channel head coil at Pennington Biomedical Research Center. Anatomical imaging acquired prior to functional trials used a 256 × 256 T1-weighted protocol with 1 mm slice thickness at 1.0 × 1.0 in-plane voxel resolution at a 10° flip angle for 176 slices. Functional imaging was performed using a single-shot gradient echo EPI sequence (TR = 2000 ms, TE = 20 ms, FOV = 22.4 cm, flip angle = 90°, bandwidth = 7812.5 Hz/px, echo spacing = 0.578 ms for 38 slices) with an in-plane voxel resolution of 3.0 mm × 3.0 mm and 3 mm slice thickness in interleaved ascending order. Functional trials were 582 volumes in length, while the functional localizer comprised 179 volumes. An initial three-volume set of dummy volumes from each run was excluded from the analysis.

#### Pre-processing and whole-brain univariate analysis

Analysis of acquired BOLD data was conducted within the FSL software package using the fMRI Expert Analysis Tool version 6.00. Anatomical data were first skull-stripped to isolate the brain from surrounding tissue using FSL’s BET module, while functional data underwent motion correction using FSL’s motion outliers function to identify and model out frame-wise displacement greater than 0.9 mm. Functional data were then motion corrected using MCFLIRT, slice-time corrected using Fourier-space time-series phase-shifting, smoothed with a Gaussian kernel of full width at half maxiumum 7 mm, and ran through a 100s high-pass temporal filtering (Gaussian-weighted least-squares straight line fitting).

The data were analysed within the General Linear Model using a multi-level mixed-effects design. The first-level analysis was carried out at the single-subject level, and each run was modelled separately. In the first-level analysis, trial-by-trial data were modelled with 6 regressors of interest corresponding to each our primary experimental conditions: single, double or triple CS + US trials, and single, double, or triple CS-alone trials. Importantly, the modelling of a given trial was based on what type of trial sequence had immediately proceeded it (see Figure 2C). For example, following two CS + US trials, the subsequent trial would be modelled as a double CS + US trial regardless of whether or not it was a CS + US or CS-alone trial itself. Additionally, in a triple CS + US sequence, the second trial in the sequence would be modelled as a single CS + US trial and the third trial in the sequence would be modelled as a double CS + US trial in the GLM, in reference to the preceding CS + US trials. Importantly, as we aimed to distinguish participants’ CR to the onset of the CS from their unconditioned response (US) to the shock itself, we adopted a strategy reported previously in the literature (Tabbert *et al.*, 2006; Klumpers *et al.*, 2010, 2017) in which the onset of the CS is modelled separately from the offset of the CS with and without the US (depending on the trial). Specifically, our regressors of interest comprised the onset of the CS and were of 5 ms in duration. We also included two important nuisance regressors to address the possibility that the US was a potential confound, one for the US delivery which began 5 ms before CS offset and was 5 ms in duration (i.e. to model out the impact of the physical shock) and one for US expectancy on non-shock trials (i.e. the same time period when shock would have occurred in a shock trial) to account for any confounding anticipatory US reactivity (Tabbert *et al.*, 2006; Dunsmoor *et al.*, 2008; Wood *et al.*, 2013). Each of the regressors of interest and the regressors relating to the US delivery and US expectancy were convolved using a double-gamma hemodynamic response function (HRF) including a model for the temporal derivative of each. We also included regressors of no-interests relating to motion, including the 6 standard and 18 extended motion correction parameters, and motion censoring regressors from the frame-wise displacement evaluation at pre-processing.

Importantly, a first-level weighted contrast was created to weight trials based on the expectancy *vs* the associative strength models, respectively. To evaluate the regions associated with the expectancy model, we assigned the highest weight (5) to triple CS-alone trials and the lowest weight (−5) to triple CS + US trials. The other weights were as follows: double CS-alone (3), double CS + US (−3), single CS-alone (1) and single CS + US (−1). Weighting conditions by ± two was done to evaluate the presence of a linear effect by ensuring that the weighted difference was equal between adjacent trial types in the contrast model (i.e. a between conditions contrast of [−5 −3 −1 1 3 5]). Thus, when performing the group analysis, comparing regions in which this contrast was greater than zero would reveal regions consistent with the expectancy strength model. Additionally, regions showing significantly less activity than zero in this same contrast would reveal those regions consistent with the associative strength model.

An additional set of univariate analyses contrasted BOLD activity during CS + US *vs* CS-alone sequences. Level 1 contrasts were generated for triple CS + US *vs* triple CS-alone, double CS + US *vs* double CS-alone, single CS + US *vs* single CS-alone trials and all CS + US *vs* all CS-alone trials.

We also contrasted the shock *vs* no-shock events at the end of each trial. This allowed us to confirm that our modelling of the end of the shock trials was successful at capturing the variance in the BOLD signal associated with the shock itself.

All first-level contrasts were then combined in second-level analyses at the single-subject level via fixed-effects analysis and transformed into MNI space. Finally, group-level analyses were performed using FLAME 1 + 2 mixed effects modelling (Worsley, 2001), with a voxel-level threshold of Z > 2.3 (one-tailed) and a corrected cluster size probability of *P* = 0.05 (Faul *et al.*, 2020; Lange *et al.*, 2020; Clewett *et al.*, 2022; Burleigh and Greening, 2023). As compared to other neuroimaging packages, FSL’s FLAME1 + 2 with a voxel threshold of Z >2.3 and cluster-correction on event-related data offers one of the most robust controls against type-1 errors (Eklund *et al.*, 2016). To further improve the spatial precision of the reported significant clusters, we have also archived our unthresholded z-stat maps at https://identifiers.org/neurovault.collection:12662.

We conducted an additional analysis using finite impulse response (FIR) modelling, rather than convolving our trial models to the hemodynamic response. This allowed for modelling the time-course of the BOLD response both during the CS presentation and after, into the inter-trial interval. Specifically, we included ten 2 sec time bins, with one bin being the duration of a TR consistent with previous literature (Andreatta *et al.*, 2015). The purpose for the FIR was two-fold. First, we could further confirm that the linear trends observed using the primary analysis above were not confounded by the presence of the shock. In particular, we predicted that the BOLD activity in the third FIR bin, corresponding to between 4 and 6 seconds after the CS onset, should reveal a pattern of activity across conditions that was qualitatively similar to the pattern observed in the primary analysis. Additionally, we predicted that we would observe a BOLD response to the shock in FIR bins 7–8, corresponding to 12–16 seconds after CS onset (i.e. 2–6 seconds after the CS offset and the delivery of the shock). Second, the FIR allowed us to perform an exploratory analysis on individual FIR bins, to evaluate whether there were potentially significant BOLD effects not captured by the primary analysis. Specifically, using the same weighted contrast approach above we analysed the fifth FIR bin, corresponding to 8–10 seconds after CS onset, which potentially captures an immediate anticipatory response to the US (Linnman *et al.*, 2011; Dunsmoor and LaBar, 2012) while remaining unconfounded by the BOLD response to shock delivery. As in the primary analysis, the group-level map was thresholded to Z > 2.3 (one-tailed) with a (corrected) cluster size probability of *P* = 0.05.

#### fMRI region-of-interest analysis

As the amygdala is often implicated in the literature on fear conditioning, we also conducted a region-of-interest analysis (ROI) on the bilateral amygdala. The amygdala ROI was created by taking only the areas of overlap between the anatomical amygdala mask from the Harvard–Oxford atlas thresholded at 50% (Greening *et al.*, 2022) and regions of bilateral amygdala significantly activated in the whole-brain Shock > No shock (Schiller *et al.*, 2013) analysis described above. The percent signal change was extracted from the ROI for each of the 6 conditions of interest for each participant and analysed using a 2 (CS-alone, CS + US) by 3 (Sequence of 1, 2, or 3) repeated measures analysis of variance (ANOVA).

## Results

### Expectancy ratings

**Fig. 3.**
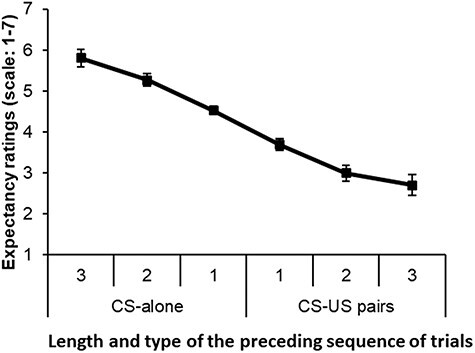
Self-reported expectancy ratings by preceding trial sequence. Error bars represent standard error of the mean.

A repeated-measures ANOVA on expectancy ratings performed with trial sequence (6 levels) as a within-subject variable revealed a significant main effect of trial sequence [*F*(5, 100) = 57.98, *P* < 0.001, $\eta _p^2$ = 0.744]. A significant linear trend was also identified, indicating that expectancy ratings decreased linearly with sequential shock [*F*(1, 20) = 71.88, *P* < 0.001, $\eta _p^2$ = 0.782] (Figure 3).

### fMRI univariate whole-brain results

The unthresholded whole-brain maps reported below, including those that were non-significant, can be found at https://identifiers.org/neurovault.collection:12662.

#### Associative strength model

There were no significant clusters responding consistent with the associative strength model (Table 1).

**Table 1.** Linear contrasts for associative strength and expectancy trends

					MNI		
#	k	Brain region	H	Z	*x*	*y*	*z*
Associative strength trend							
		None					
Expectancy trend							
1	3946	Intracalcarine cortex, cuneal cortex, precuneus cortex, super division of lateral occipital gyrus, angular gyrus (L)	R/L	3.46	−30	−84	38
2	2768	Temporal fusiform cortex, temporal occipital fusiform cortex, parahippocampal gyrus, inferior temporal gyrus, middle temporal gyrus, cerebellum	R	3.57	26	−56	−18
3	1245	Middle frontal gyrus, superior frontal gyrus	L	3.4	−30	22	60

# = the number of a cluster, ordered by size; k = the number of contiguous voxels in the cluster; Brain region = regions of local maxima included in the broader cluster. The region names are taken generally from the Harvard Oxford atlas in FSL; H = principal hemisphere of the cluster, right (R) or left (L); Z = maximum z-value from the cluster within the given brain region; MNI(X, Y,Z) = coordinates of the voxel with the maximum effect in the standardized space of the Montreal Neurological Institute (MNI), represented in units of millimeters (mm).

#### Expectancy model

**Fig. 4.**
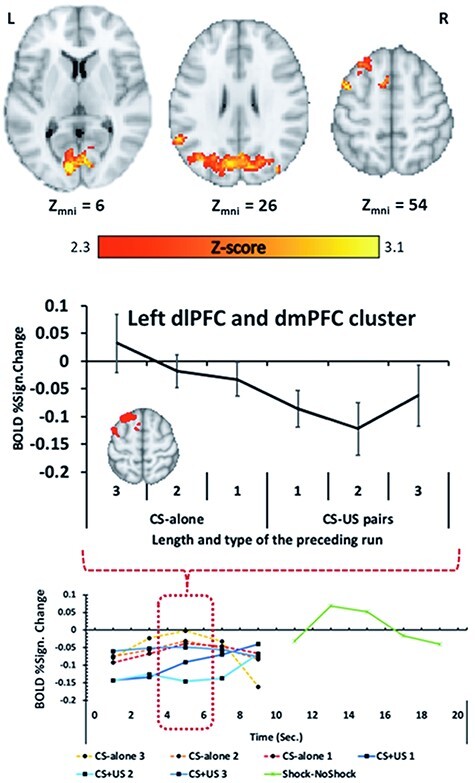
TOP—BOLD activity identified in the whole-brain linear contrast analysis, in which brain activity displaying a linear trend consistent with the expectancy model is shown in red-yellow. The clusters displayed were thresholded at the voxel level and corrected for multiple comparisons. MIDDLE—A graphical visualization of BOLD activity in the left dlPFC from the linear contrast analysis. It reveals a trend consistent with the expectancy model. Error bars represent standard error of the mean. BOTTOM—FIR results extracted from the dlPFC and dmPFC cluster. The dashed-red line highlights FIR bin 3 corresponding to 4–6 seconds following CS onset and the pattern of effects consistent with the results from the primary analysis and the expectancy model. The unthresholded whole-brain maps can be found at https://identifiers.org/neurovault.collection:12662).

The expectancy trend analysis shows a network of regions responding consistent with the expectancy model (Figure 4, Orange; Table 1). Longer sets of sequential CS-alone trials were followed by elevated activity in aspects of the left dlPFC and left dmPFC (Figure 4—Middle) including parts of the middle frontal gyrus and superior frontal gyrus, as well as the left angular gyrus. In the right hemisphere, there was significant activation in the parahippocampal gyrus and inferior temporal gyrus. This analysis also identified activity in bilateral aspects of the visual cortices including the cuneal cortex and the lateral occipital gyrus.

The results of the FIR analysis (Figure 4—Bottom) further confirm the expectancy trend from the results above and rule out the possibility that the presence of the shock was confounding the primary analysis. Specifically, after extracting and plotting the mean FIR responses in the left dlPFC and dmPFC from the primary analysis, we observed that there was a linear trend consistent with the expectancy model 4–6 seconds following CS onset, similar to the results of the primary analysis (Figure 4—Middle). We also observed a BOLD response to the US (shock-no shock) occurring 12–16 seconds after CS onset (i.e. 2–6 seconds following CS offset) in left dlPFC and dmPFC (Figure 4—Bottom).

#### Contrasts of CS-alone and CS + US

To further clarify the neural regions associated with sequences of CS-alone *vs* the CS + US, an additional set of exploratory univariate whole-brain analyses were conducted (see Table 2 for full results).

**Table 2.** Simple contrasts

					MNI		
#	k	Brain region	H	Z	*x*	*y*	*z*
Single CS alone > single CS + US							
1	33 799	Inferior frontal gyrus, anterior cingulate gyrus, precentral gyrus, post-central gyrus, middle frontal gyrus, middle temporal gyrus, inferior temporal gyrus, supramarginal gyrus, superior parietal lobule, lateral occipital cortex, insula (L), cerebellum	R/L	4.58	52	6	36
Double CS alone > double CS + US							
1	4598	Temporal fusiform cortex, temporal occipital fusiform cortex, brain stem	R/L	3.38	4	−50	−22
2	2275	Middle frontal gyrus, superior frontal gyrus	R/L	3.5	−6	12	66
Triple CS alone > triple CS + US							
1	1507	Cuneal cortex, intracalcarine cortex, precuneus	R/L	3.2	24	−78	38
Single CS + US > single CS alone							
		None					
Double CS + US > double CS alone							
		None					
Triple CS + US > triple CS alone							
		None					
All CS alone > all CS + US							
1	22 631	Intracalcarine cortex, cuneal cortex, precuneus cortex, super division of lateral occipital gyrus, temporal fusiform cortex, temporal occipital fusiform cortex, parahippocampal gyrus, inferior temporal gyrus, middle temporal gyrus, cerebellum, brain stem, middle frontal gyrus (L), superior frontal gyrus (L), angular gyrus (L)	R/L	3.83	10	−70	−26
All CS + US > all CS alone							
		None					

# = the number of a cluster, ordered by size; k = the number of contiguous voxels in the cluster; Brain region = regions of local maxima included in the broader cluster. The region names are taken generally from the Harvard Oxford atlas in FSL; H = principal hemisphere of the cluster, right (R) or left (L); Z = maximum z-value from the cluster within the given brain region; MNI(X, Y,Z) = coordinates of the voxel with the maximum effect in the standardized space of the Montreal Neurological Institute (MNI), represented in units of millimeters (mm).

*CS + US > CS-alone:* We observed no significant clusters of activity across any of the contrasts associated with CS + US > CS-alone.

*CS-alone > CS + US:* In the single CS-alone > single CS + US condition, we identified a single large cluster that included several bilateral regions. These regions included aspects of the prefrontal cortex including the inferior frontal gyrus, anterior cingulate gyrus and middle frontal gyrus. This cluster also included an aspect of the left insula.

In the double CS-alone > double CS + US trials, we observed elevated activation in parts of bilateral dlPFC including the middle frontal and superior frontal gyri, as well as the bilateral fusiform cortex and brain stem.

In the triple CS-alone > triple CS + US contrast, significantly elevated activity was found only in bilateral aspects of the early visual cortex including the cuneal and intracalcarine cortices, as well as the precuneus.

Lastly, we compared the CS-alone all trials > CS + US all trials. This revealed robust activity in a single cluster that spanned several regions. Notably, this analysis revealed bilateral activation in parts of the early visual cortex, including the intracalcarine and cuneal cortices, in parts of late visual regions such as the lateral occipital gyrus and the fusiform cortices. We also observed significant activation in aspects of left dlPFC in parts of the middle frontal and superior frontal cortices.

#### Contrast of Shock vs No-shock

In a final analysis, we ensured that our shock *vs* no shock modelling of the end of trials was successful in capturing the variance in activity associated with the shock *vs* no-shock (for full results see Table 3).

**Table 3.** Whole-brain contrast of Shock *vs* No Shock

					MNI		
#	k	Brain region	H	Z	*x*	*y*	*z*
Shock > No Shock							
1	71 060	Amygdala, hippocampus, insula, anterior cingulate gyrus, inferior frontal gyrus, post-central gyrus, middle temporal gyrus, supramarginal gyrus, lateral occipital cortex (L), cuneal cortex, precuneus cortex, thalamus	R/L	5.72	−54	−26	20
2	1502	Lateral occipital cortex	R	5.72	36	−80	−20
No Shock > Shock							
1	2814	Anterior cingulate gyrus, frontal medial cortex, frontal pole, paracingulate gyrus, subcallosal cortex	R/L	4.67	−10	46	−14

# = the number of a cluster, ordered by size; k = the number of contiguous voxels in the cluster; Brain region = regions of local maxima included in the broader cluster. The region names are taken generally from the Harvard Oxford atlas in FSL; H = principal hemisphere of the cluster, right (R) or left (L); Z = maximum z-value from the cluster within the given brain region; MNI(X, Y,Z) = coordinates of the voxel with the maximum effect in the standardized space of the Montreal Neurological Institute (MNI), represented in units of millimeters (mm).

**Fig. 5.**
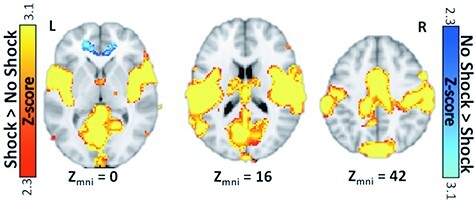
BOLD activity identified by whole-brain, corrected, Shock versus No Shock trials. Red-Yellow clusters represent Shock > No Shock trials. Blue clusters represent No Shock > Shock trials. The clusters displayed were thresholded at the voxel level and corrected for multiple comparisons. The unthresholded whole-brain map can be found at https://identifiers.org/neurovault.collection:12662).

Shock > No-shock: We observed significant bilateral activation across a range of regions associated with somatosensation and pain or aversion. Most notably, we observed activation of bilateral post-central gyrus, dorsal insula and the amygdala (Figure 5).

No-shock > Shock: We also observed significant activity in one cluster during no-shock trials. The cluster was found in the medial prefrontal cortex including parts of the anterior cingulate gyrus, frontal medial cortex and subcallosal cortex (Figure 5).

### fMRI univariate ROI results

No significant effects were observed in the bilateral amygdala ROI.

### FMRI exploratory FIR results

The results of a whole-brain analysis on the fifth FIR bin, corresponding to 8–10 seconds after CS onset, revealed several clusters of activity consistent with the associative strength model (Figure 6; Table 4). Notably, there was significant activity in the left insula, including the parts of both the anterior and posterior insula, and the left visual cortex, including the occipital pole. Additionally, activity during this time-window of the FIR was also not confounded by the shock delivery, as shock-related BOLD response to shock-no shock was observed in FIR bins 7–8 (2–6 seconds after CS offset) as can be seen in Figure 6.

**Table 4.** Exploratory whole-brain results from the fifth FIR bin

					MNI		
#	k	Brain region	H	Z	*x*	*y*	*z*
Associative strength trend							
1	1441	Insula, parietal operculum, post-central gyrus, precentral gyrus, superior temporal gyrus, supramarginal gyrus	R	3.58	56	−6	14
2	1074	Lateral occipital cortex, occipital pole	R	3.58	12	−100	16
Expectancy trend							
		None					

# = the number of a cluster, ordered by size; k = the number of contiguous voxels in the cluster; Brain region = regions of local maxima included in the broader cluster. The region names are taken generally from the Harvard Oxford atlas in FSL; H = principal hemisphere of the cluster, right (R) or left (L); Z = maximum z-value from the cluster within the given brain region; MNI(X, Y,Z) = coordinates of the voxel with the maximum effect in the standardized space of the Montreal Neurological Institute (MNI), represented in units of millimeters (mm).

**Fig. 6.**
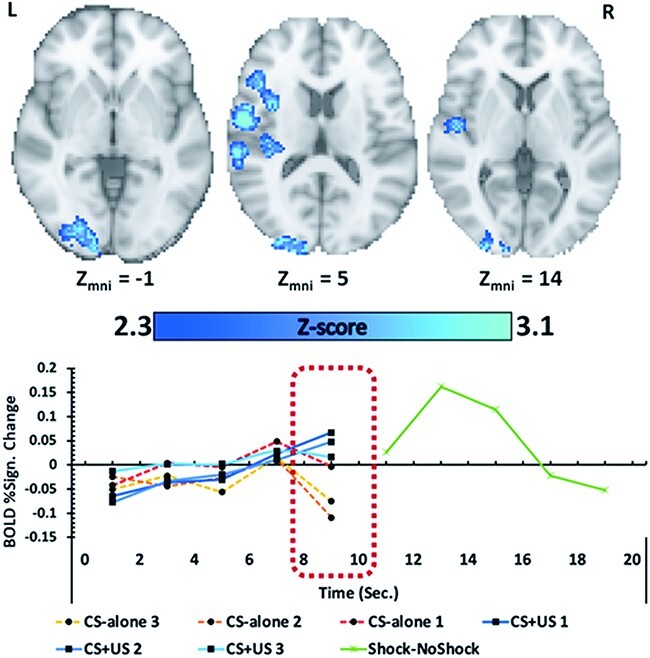
TOP—Whole-brain results of the exploratory FIR analysis involving the 5th FIR bin corresponding to 8–10 seconds from CS onset (i.e. proximate to CS offset, at 10 seconds). The regions shown in blue-light blue responded with a linear trend consistent with the associative strength model. BOTTOM—The mean BOLD response was extracted from the insula cluster to plot the full range of FIRs. The dashed-red line highlights the 5th FIR bin and the pattern of results relating to the associative strength model.

## Discussion

This study tested the prediction that there are dissociable brain networks reflecting the associative strength model and the expectancy model of Pavlovian fear conditioning, consistent with two-system theories of threat and fear reactivity. We observed that trial-by-trial self-reported expectancy trended negatively with repeated CS + US pairings consistent with the expectancy model prediction. Neurologically, we observed a significant linear increase in parts of the left dlPFC and the left angular gyrus, as well as parts of the right parahippocampal gyrus and bilateral aspects of the visual cortices as the number of sequential CS-alone trials increased consistent with the expectancy model. On the other hand, we observed no significant linear effect consistent with the associative strength model in our primary analysis, nor did we observe any significant activity in brain regions associated with significantly greater activity during CS + US trials compared to CS-alone trials in any of the simple contrasts we ran. Additionally, we observed no significant effects in the bilateral amygdala ROI analysis. Nevertheless, in an exploratory FIR analysis, we did observe a significant linear trend consistent with the associative strength model in the BOLD signal produced near the end of the CS presentation, closer in time to the ‘anticipate’ presentation of the US. This analysis revealed a significant increase in activity in the insula and early visual cortex as the number of sequential CS + US trials increased. Overall, rather than our findings being consistent with a two-system model of fear processing, the results of our primary analysis highlight both the behavioural and neural correlates associated with the expectancy model of associative learning.

Self-reported expectancy ratings in this study were consistent with the expectancy model. We observed a significant linear effect such that participants’ expectation of receiving shock increased with the number of sequential CS-alone trials and decreased with the number of sequential CS + US trials. This is consistent with previous research using similar paradigms (Perruchet, 1985; Moratti and Keil, 2009), as well as the gambler’s fallacy (Burns and Corpus, 2004). Our findings were distinct from several studies using the Perruchet paradigm (Perruchet, 2015) which have reported that CRs parallel associative strength when measuring somatic (Weidemann *et al.*, 2012) and autonomic (Perruchet *et al.*, 2015) CRs, as well as in cued reaction time tasks (Perruchet *et al.*, 2006). Therefore, while self-report findings in the present study reflected the expectancy theory, future research could benefit from the inclusion of behavioural or peripheral physiological measures, which may reflect the associative strength model (Perruchet *et al.*, 2006).

Along with the behavioural findings, we also observed a wide network of regions displaying a linear relationship consistent with the expectancy model. Most notably, activity was observed in a cluster that included aspects of both the left dlPFC and left dmPFC including the middle frontal and superior frontal gyri. Consistent with the present findings and the expectancy theory, the dlPFC appears to be a key region in both the gambler’s fallacy (Xue *et al.*, 2011, 2012) and participant’s contingency knowledge with respect to the CS and US (Carter *et al.*, 2006). Complementary to the dlPFC, the dmPFC has also been implicated in conscious, descriptive elements of fear conditioning, most notably in instructed fear conditioning in which the CS+ is never physical paired with the US (Mechias *et al.*, 2010; Maier *et al.*, 2012). Other research has observed that both the dlPFC and dmPFC are involved in the trial-by-trial generation of threat anticipation (Greening *et al.*, 2016), though this study used trace as opposed to delay conditioning. Additionally, at least one brain lesion study involving fear conditioning found that the dmPFC and dlPFC are not required for the acquisition of differential fear conditioning, though they are involved in conscious modulation of fear or threat expression (Kroes *et al.*, 2019). It should also be noted, two more recent meta-analyses (Fullana *et al.*, 2016, 2018) of fear conditioning observed aspects of both dlPFC and dmPFC involvement in differential fear conditioning (i.e. not just instructed conditioning). Most studies included in these meta-analyses used partial reinforcement schedules in which the potential contributions of expectancy *vs* associative strength are not isolated from one another. Taken together, the activation of these dorsal regions of the PFC appears to underscore the potential relationship between higher-order cognitive processes such as conscious awareness and expectation, and the expression of fear conditioning (Mitchell *et al.*, 2009) or the subjective experience of fear (Taschereau-Dumouchel *et al.*, 2019).

Additionally, we observed significant activity in clusters that included the right parahippocampal and inferior temporal gyri that trended positively with expectancy. Previous research using differential fear conditioning finds that activation of aspects of the medial temporal lobe, such as the parahippocampal gyrus, is associated with contingency awareness (Carter *et al.*, 2006). This is consistent with earlier studies of brain lesions patients revealing that lesions affecting the medial temporal lobe display impaired declarative knowledge of the contingency between the CS and US, though there is preserved evidence of an implicit associative memory (Bechara *et al.*, 1995; Clark and Squire, 1998). Alternatively, previous research into fear generalization finds that activity in parts of the medial temporal lobe and precuneus cortex linearly increases as generalized conditioned stimuli become perceptually more similar to the CS-, which has been interpreted as safety-related activity (Lissek *et al.*, 2013; Lange *et al.*, 2017). Thus, it is possible that both the parahippocampal and precuneus activity observed in the present study reflects a safety signal. However, this interpretation is inconsistent with our self-report behavioural data regarding shock anticipation.

Regarding the early visual cortex, we observed medial and lateral visual regions associated with expectancy. This is distinct from Moratti and Keil (2009) in which visual cortex activity appeared to reflect the associative strength theory. Several potential factors may have contributed to this difference. First, the properties of the stimulus chosen as the CS may have influenced where the impact of associative strength occurred. Moratti and Keil (2009) used a basic visual grating (i.e. a 45-degree contrast grating) as their CS with a flickering luminance at 12.5 Hz. This low-level stimulus-type is primarily processed in the primary visual cortex, which has been shown to respond to a low-level visual stimulus undergoing fear conditioning (Padmala and Pessoa, 2008; McTeague *et al.*, 2015). Conversely, we used a static human face image. Faces are preferentially processed in a more holistic manner later in the ventral visual stream, primarily in the right lateral occipital cortex and right fusiform cortex (Grill-Spector *et al.*, 2001). Second, our stimulus is distinct from Moratti and Keil (2009) in terms of biological relevance to fear or threat (i.e. fear-relevant stimuli). Fear-relevant stimuli such as faces (or snakes and spiders) have been found to fear condition more readily and extinguish more slowly compared either fear-irrelevant stimuli such as flowers, mushrooms or artifactual images (Mineka and Öhman, 2002; Atlas and Phelps, 2018). However, findings regarding the relationship between fear-relevant stimuli and rates of conditioning and extinction are mixed (Åhs *et al.*, 2018). A final consideration is the temporal differences between the two distinct imaging modalities that were used. Whereas Moratti and Keil (2009) examine steady-state evoked potentials in magnetoencephalography (MEG), we used fMRI which has a much slower temporal resolution. One possibility is that the relative contribution of early (or bottom-up) *vs* later (or top-down) input to the early visual regions is differentially quantified by each imaging technique. For example, the visual cortex activity revealed by Moratti and Keil (2009) might reflect relatively earlier signals (Gordon *et al.*, 2017) generated from more automatic subcortical circuits associated with defensive responding (Moratti *et al.*, 2006). On the other hand, the visual cortex findings of the present study may reflect relatively later and sustained conscious attentional processes, which might be related to activation also observed in aspect of the dorsal PFC and angular gyrus. Also related to the early visual findings, previous research by Tabbert *et al.* (2006) suggests that greater visual cortex activity is observed for participants who are unaware of the contingency between the CS+ and US. It is unlikely that unawareness is contributing to our findings given that we used a single-cue conditioning paradigm in which all participants, according to the behavioural data, were aware to some degree that the CS was predictive of the US.

Complicating the above discussion are the results of our exploratory FIR analysis using the 5th time bin (8–10) seconds following CS onset. This analysis revealed significant activity in both the right insula and early visual cortex consistent with the associative strength model. This later time window potentially reflects processes relating to the immediate anticipation of the US (Linnman *et al.*, 2011; Dunsmoor and LaBar, 2012). This finding suggests the possibility that there are two-systems for threat and fear processing, one relating to conscious expectations and the other relating to the strength of the association between a CS and US, which might show differential responses depending on timing. Such timing effects could include the time from the CS onset and the temporal proximity to the US. However, given this was an exploratory analysis, the results should be interpreted with caution, and future research might consider effects relating to the CS *vs* US anticipation in the context of the associative strength *vs* expectancy model.

Given the sample size of the present study, an additional consideration is that the lack of whole-brain findings particularly for the associative strength model could reflect low power (Cremers *et al.*, 2017). Unfortunately, there is presently no agreed upon method or stable software for conducting power analyses for whole-brain fMRI, as even the most notable options are presently defunct or unavailable (Joyce and Hayasaka, 2012; Mumford, 2012; Durnez *et al.*, 2016). Nevertheless, for full transparency of reporting, we have also provided each of the unthresholded whole-brain maps (https://identifiers.org/neurovault.collection:12662), which could facilitate effect size estimates on the present study in future meta-analyses or for estimating sample sizes in future studies.

It should also be noted that our study used a single-cue conditioning paradigm, consistent with the previous behavioural (Perruchet, 1985; Perruchet *et al.*, 2015) and MEG (Moratti and Keil, 2009; Yuan *et al.*, 2018) studies that were conducted using the Perruchet paradigm. This contrasts with much of the research involving brain lesion patients (Bechara *et al.*, 1995; Clark and Squire, 1998), and both uninstructed (Fullana *et al.*, 2016) and instructed fear conditioning (Mechias *et al.*, 2010) experiments in fMRI, which uses differential fear conditioning paradigms involving both a CS+ and CS-. Future research could evaluate whether the presence of a safe conditioned stimulus (i.e. CS-) and use of differential conditioning affect associative strength, as single cue conditioning may be more automatically acquired (Carter *et al.*, 2003) and may be associated with patterns of brain activity that are distinct from those of differential conditioning (Cheng *et al.*, 2003). In addition, due to the nature of the single-cue paradigm used in the present study, which was adopted from Perruchet (1985), we needed to include trials in which the US (i.e. the mild shock) was also delivered. Although it is common in fMRI studies of fear conditioning to exclude trials containing the US, there are some studies that have analysed the CRs to trials in which the US is present using methodological approaches that were used in the present study. Consistent with these latter studies, the present study used a relatively lengthy delay between the onset of the CS and the onset of the US (Tabbert *et al.*, 2005; Dunsmoor *et al.*, 2008), which was 9.95 seconds in the present study, and independently modelled the CS, US (i.e. shock) and no-US (i.e. no-shock) events per trial (Tabbert *et al.*, 2005; Klumpers *et al.*, 2010). The analyses of the shock > no-shock also revealed a pattern of robust activation consistent the previous literature examining unconditioned responses to shock *vs* no-shock (Tabbert *et al.*, 2005; Linnman *et al.*, 2011) and to aversive stimuli more generally (Xu *et al.*, 2020). This pattern of activation included bilateral regions of the amygdala, insula, anterior cingulate gyrus and somatosensory regions. Lastly, a follow-up FIR analysis confirmed that the linear trend consistent with the expectancy model was present around 4–6 seconds following the CS onset and was not confounded by the shock effect. Together, it appears that our modelling of the shock *vs* no-shock was successful, and it is unlikely that our findings regarding the brain-based CRs were confounded by the shock.

Although the findings of the present study do not directly support two-system models of fear such as that proposed by LeDoux and Pine (2016), in combination with many of the findings cited above, a two-system model might best reflect how the brain forms and expresses fear conditioning. LeDoux and Pine differentiate changes induced by associative learning from those related to the conscious state of fear and anxiety. This two-system view poses that both systems can be active simultaneously but targeted separately for analysis or intervention. While our expectancy network may be considered a component of conscious ‘feeling states’ relevant to fear, other regions more commonly implicated in the ‘defensive responding’ circuit such as the amygdala might respond consistent with the associative strength model. However, future research is required to examine further which brain regions respond consistent with the associative strength model given the lack of such findings in the current study.

## Data Availability

The data underlying this article will be shared on reasonable request to the corresponding author.
